# An in-vivo study of the safety of copper-containing intrauterine devices in 3.0 Tesla magnetic resonance imaging

**DOI:** 10.1007/s00261-024-04493-4

**Published:** 2024-07-18

**Authors:** Jeffrey Moy, Matthew Landon, John Vigilante, Benjamin Lehmann, Amber DeChambeau, Frederick Rohlfing, Robert Marks

**Affiliations:** 1 Department of Radiology, Naval Medical Center San Diego, 34800 Bob Wilson Drive, Suite 204, San Diego, CA 92134 USA; 2 Department of Radiology, Sharp Rees-Stealy Medical Group, 300 Fir Street, San Diego, CA 92101 USA; 3 Department of Radiology, University of California San Diego, 200 West Arbor Drive, San Diego, CA 92103 USA

**Keywords:** Magnetic resonance imaging, Intrauterine device, Paragard T380A, Copper IUD, MRI safety

## Abstract

**Purpose:**

The aim of this study is to prospectively evaluate whether women with copper-containing intrauterine devices (Cu-IUD), currently listed as MR conditional, can safely undergo 3.0 Tesla (3 T) magnetic resonance imaging (MRI).

**Methods:**

73 women, age 18–54 years old, with a Cu-IUD who were undergoing MRI for any reason were included consecutively. Pre- and post-MRI standard pelvic ultrasound examinations were completed to determine the appropriate pre- and post-MRI positioning of the Cu-IUD. Displaced IUDs were defined by IUD crossbars not in the fundal portion of the endometrial cavity, a visualized tip in the mid or lower uterus, any part of the device located in the cervical canal or outside of the endometrial canal, a fractured device, or a non-visualized IUD. Additionally, a questionnaire was completed by participants to determine the level of pre- and post-MRI pelvic pain.

**Results:**

There were zero observed displaced Cu-IUDs on post-MRI pelvic ultrasounds (p = 0/70, 95% CI 0, .043). Three participants were dropped from the study due to malpositioned IUDs on pre-MRI pelvic ultrasound. Six patients reported new or worsening pelvic pain/discomfort during or after their MRI examination.

**Conclusion:**

Our results suggest that performing 3 T MRI using a low SAR setting does not cause displacement of Cu-IUDs, with zero out of 70 patients demonstrating IUD displacement.

**Graphical Abstract:**

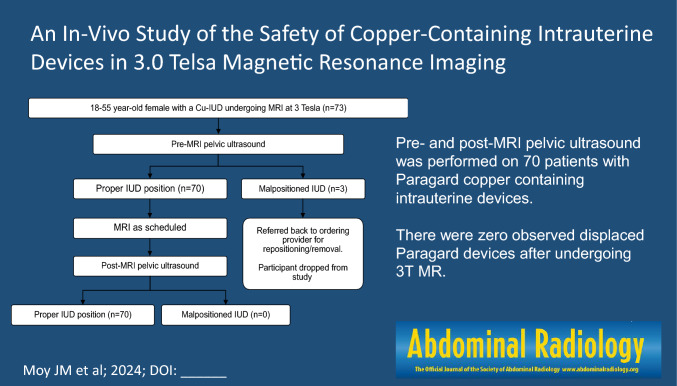

## Introduction

Intrauterine devices are long-acting reversible methods for contraception that provide high rates of contraceptive efficacy and rates of satisfaction compared to other contraceptive methods [1–4]. Copper-containing intrauterine devices (Cu-IUDs) such as the Paragard T380A, the only copper-containing and 100% hormone free IUD approved in the U.S., have shown excellent contraceptive effectiveness, despite the lack of hormones, and few contraindications [3, 5–8]. A 2006 systematic review of over 50,000 women compared various Cu-IUDs and found that those with 380 mm^2^ surface area of exposed copper were the most effective for contraception [9]. Cu-IUDs have been recommended as the first line contraceptive methods offered in women with contraindications to estrogen and/or progesterone and for patients with certain cancers, such as breast and cervical cancer [10]. Cu-IUD is also the most effective option for emergency contraception with a 92–99% effectiveness [9–11].

MRI is a common imaging modality for multiple clinical problems ranging from musculoskeletal injuries, to evaluating for and staging cancers of the bone, breast, abdomen and pelvis, chest, and nervous system. Multiple in-vitro and in-vivo studies have shown that 1.5 T MRI can be safely performed on patients with Cu-IUDs when measuring device heating, deflection, and torque [12–14]. In addition, previous in-vitro studies have shown that there are no contraindications or safety concerns for performing 3 T MRI on patients with Cu-IUDs when testing for device deflection, torque, heating and artifact [15, 16]. However, in the only in-vivo study to date of Cu-IUDs at 3 T, one of 18 included women had partial perforation and dislocation of the IUD into the fundal myometrium [17]. Although the authors acknowledged that the perforation may have occurred prior to the MRI examination, they concluded that women with Cu-IUDs should consult their healthcare professionals after an MRI at 3 T to check the correct positioning of the IUD to exclude any complications. Currently, Cu-IUDs are classified as “MR conditional”, meaning patients can be safely scanned in an MRI system when meeting specified conditions and specific absorption rate (SAR) limitations. For Paragard T380A, the only approved Cu-IUD in the United States, these conditions are a static magnetic field of 3 T or 1.5 T, maximum spatial field gradients of 4000 gauss/cm, and maximum MR system reported whole body average SAR of 2 W/kg [1].

Given the increased use of 3 T MRI as well as IUDs, it is important to further investigate the safety profile of Cu-IUDs at 3 T [18, 19]. The goal of this study is to prospectively evaluate whether patients with Paragard T380A, a Cu-IUD, could safely undergo 3 T MRI.

### Methods

#### Patients

This study was approved by the institutional review board at Naval Medical Center San Diego and was compliant with the Health Insurance Portability and Accountability Act. All patients provided written informed consent. This prospective single-site study enrolled patients who presented for 3 T MRI for any indication and had a Paragard T380A Cu-IUD between 12 January 2018 and 19 December 2019. Eligible participants were women ≥ 18 years old with a confirmed Cu-IUD who underwent 3 T MRI for any reason. Women who did not consent or were under the age of 18 years old were excluded from the study.

#### Pre-MRI ultrasound imaging

**Fig. 1:**
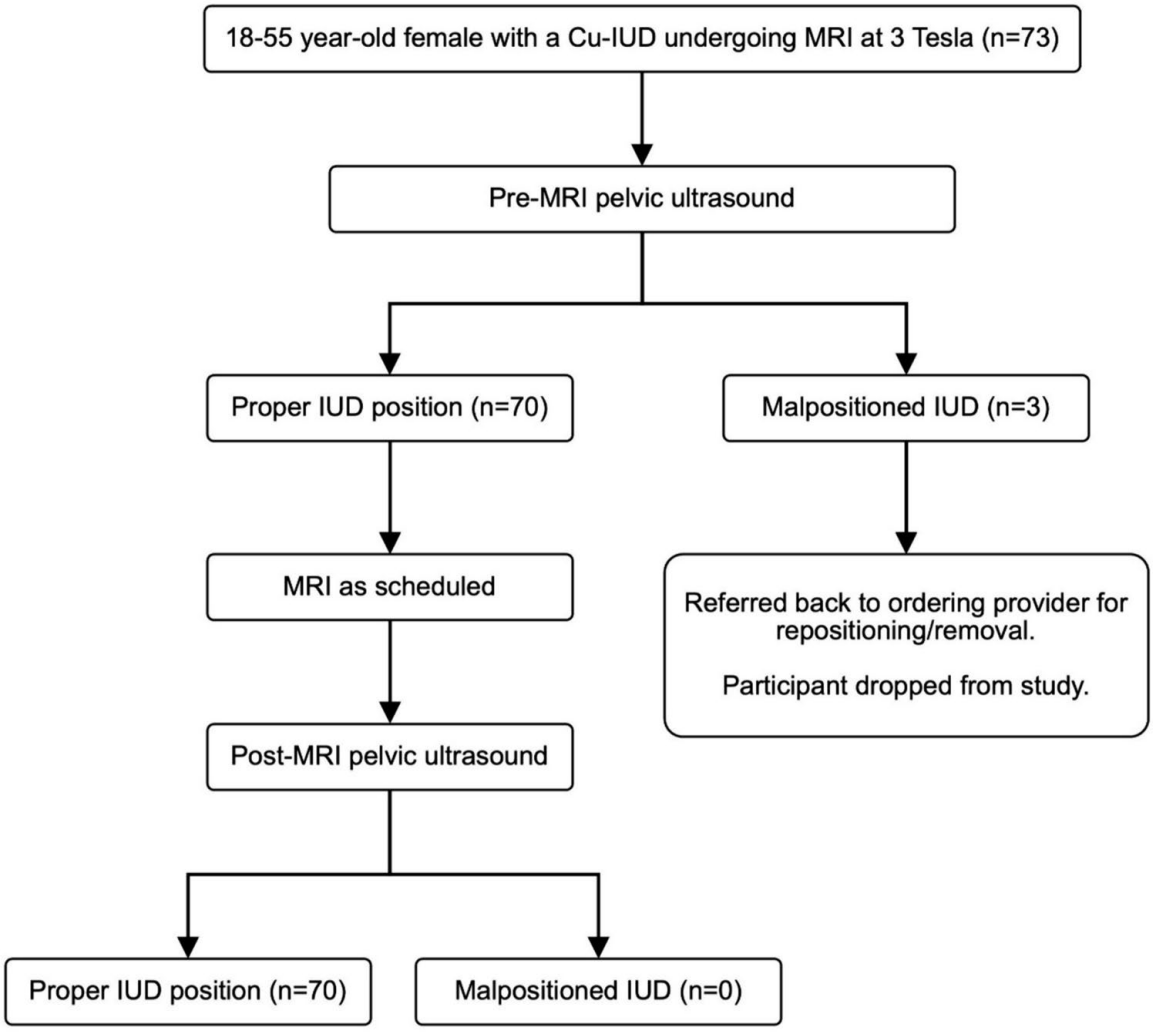
Study schema. Intrauterine device (IUD). Copper-containing IUD (Cu-IUD). Magnetic Resonance Imaging (MRI)

**Fig. 2:**
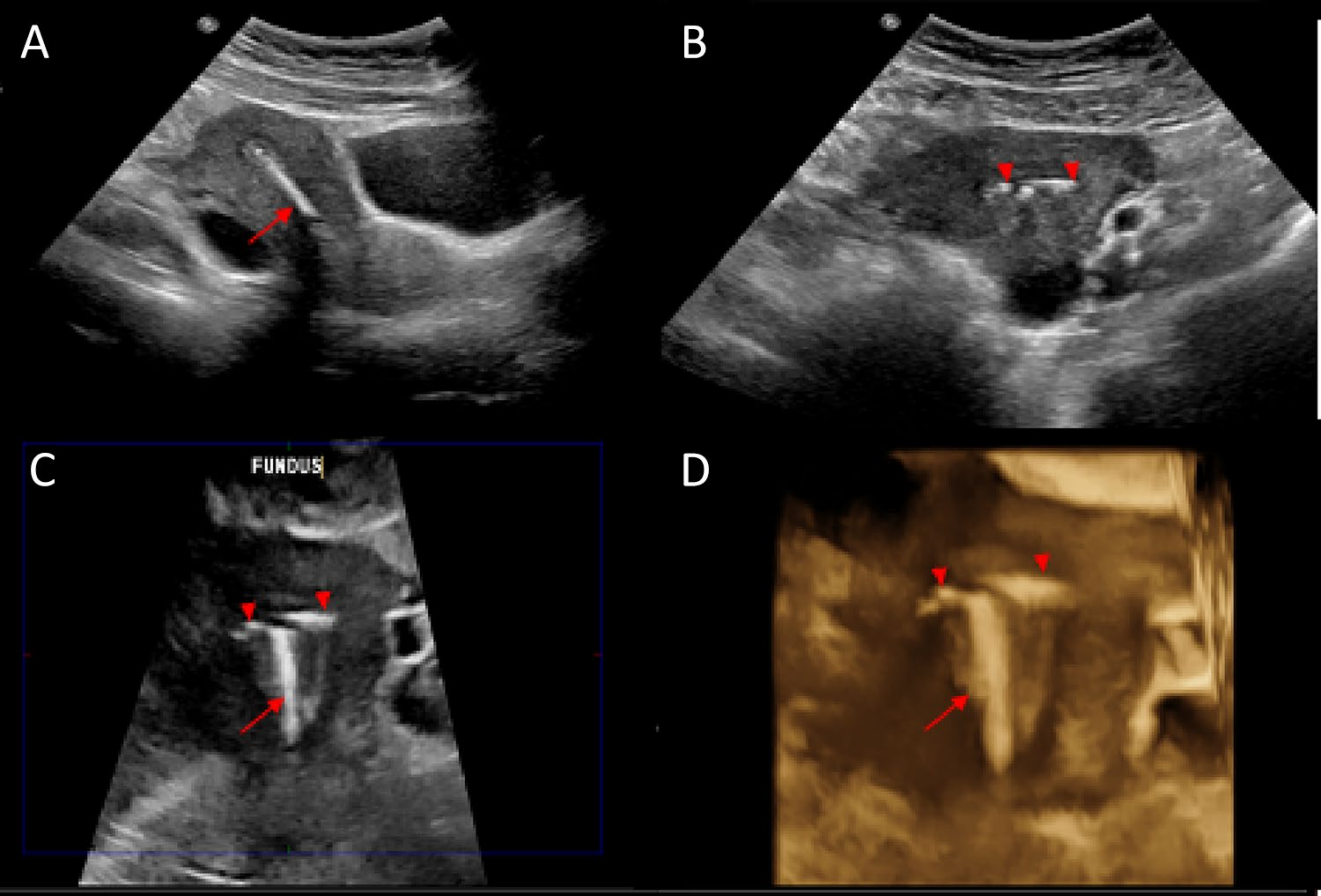
Proper IUD placement. 2D grayscale transabdominal ultrasound images of the uterus in long (**A**) and short axis (**B**) demonstrating an IUD stem (arrow) centered within the endometrial cavity and both arms (arrowheads) of the device extending laterally at the uterine fundus. 3D grayscale (**C**) and gynecologic tissue rendered (**D**) ultrasound images of the same patient confirming appropriate positioning of the stem and arms of the IUD

**Fig. 3:**
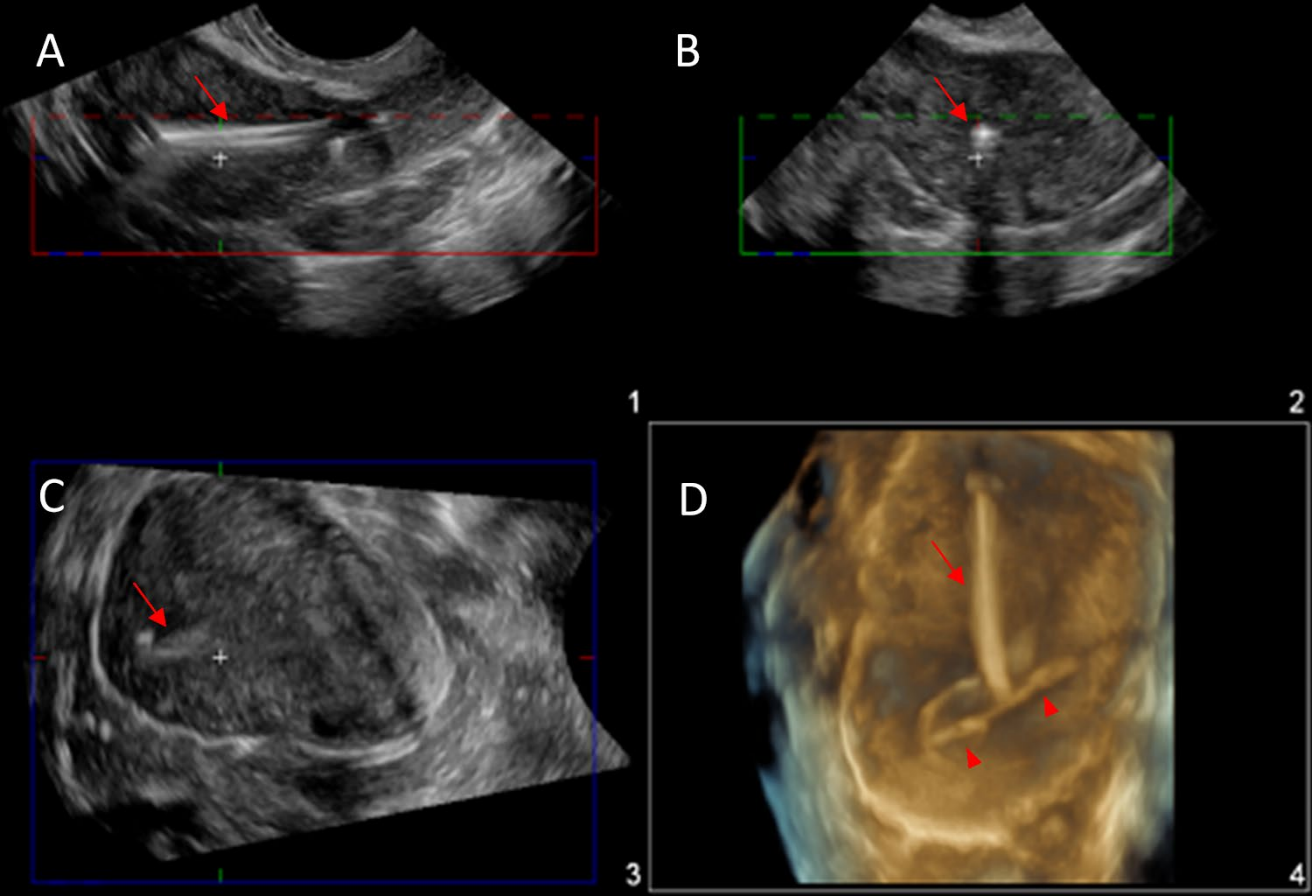
Malpositioned IUD. 2D grayscale transvaginal ultrasound images of the uterus in long (**A**) and short axis (**B**) demonstrating an IUD stem (arrow) centered within the endometrial cavity but arms not at the uterine fundus. 3D grayscale (**C**) and gynecologic tissue rendered (**D**) ultrasound images of the same patient demonstrating a flipped device with the IUD arms (arrowheads) extending outward within the lower uterus near the internal cervical os

On the day of the MRI examination, all consented patients underwent an ultrasound prior to the MRI to determine the position of the IUD (Fig. 1). All pelvic ultrasounds were performed transabdominally using Philips EPIQ 7G (Philips Healthcare, Best, Netherlands) by dedicated study sonographers. Transabdominal ultrasounds were reviewed by two study radiologists to confirm proper placement, as defined as IUD crossbar in the fundal portion of the endometrial cavity using 3D imaging (Fig. 2). A malpositioned IUD was defined as IUD crossbars not in the fundal portion of the endometrial cavity (Fig. 3), a visualized tip in the mid or lower uterus, any part of the device located in the cervical canal or outside of the endometrial canal, a fractured device, or a non-visualized IUD [20]. Per protocol, if the transabdominal ultrasound could not determine the location or position of the IUD, a transvaginal ultrasound was performed. During the study, transvaginal ultrasound was performed on one participant prior to the MRI. This additional step was performed as the IUD was found to be appropriately positioned on the transabdominal ultrasound and the sonographer performed a transvaginal ultrasound as per clinical practice, and not per protocol. If the IUD was in the proper position, then the patient was taken to the MRI suite for their clinical MRI (Fig. 1). If the IUD was malpositioned, the patient received their clinical MRI as scheduled but were removed from the study (Fig. 1). In addition, these patients were counseled to see their provider for repositioning of the IUD and to use another form of contraception in the meantime.

#### MRI imaging

All clinical MRI examinations were performed on Philips 3.0 T Ingenia (Philips Healthcare, Best, Netherlands). All MRI examinations used a “low SAR” setting to comply with the conditions for imaging patients with a Cu-IUD at 3 T [21]. The type of MRI by body part was recorded for each patient.

#### Post-MRI questionnaire and ultrasound imaging

After the MRI examination and while in the MRI suite, all patients completed a questionnaire asking about any symptoms they had during their MRI examination (Appendix 1). Patients were then sent to the ultrasound suite for a post-MRI ultrasound examination to evaluate IUD placement. IUD displacement criteria included IUD crossbars no longer positioned within the fundal portion of the endometrial cavity compared to the pre-MRI ultrasound using 3D ultrasound. Two study radiologists confirmed the location of the IUD after the MRI. Patients that demonstrated displacement of the IUD were referred to their referring physician for replacement of the IUD and counseled to use another form of contraception until discussion with their referring physician (Fig. 1).

### Statistical analysis

The data collected included the patient age, body part being imaged by MRI, and the presence or absence of pelvic pain before or after the study. The primary data point was the absence or presence of IUD displacement by post-MRI pelvic ultrasound analysis, as compared to pre-MRI pelvic ultrasound. The secondary data point was new or worsening pelvic pain, or any other new non-pelvic pain after the MRI examination.

As the only prior in-vivo study to date of Cu-IUDs at 3 T included 18 women, our study aimed to enroll between 50 and 100 patients to have over 2–5 times the number of patients in the first study [17]. The upper 95% confidence limit for p when no events were observed was calculated using the 3/n approximation as described by Hanley and Lippman-Hand [22].

## Results

A total of 73 patients with a Paragard T380A Cu-IUD enrolled in the study (Fig. 1). The mean age was 30.5 years old. Three patients were disenrolled from the study due to having malpositioned IUDs on pre-MRI ultrasound evaluation. There were zero observed displaced Cu-IUDs on post-MRI pelvic ultrasounds (p = 0/70, 95% CI 0, 0.043) [22]. Patients received MRIs of the head/neck (29%), lower extremity (23%), spine (21%), upper extremity (19%), and chest/abdomen/pelvis (9%; Table 1).

**Table 1:** Type of MRI image by age

Age (years old)	H/N	C/A/P	Spine	UE	LE
18–29	8	1	10	6	9
30–41	9	5	5	6	6
42–54	3	0	0	1	1
Total	20	6	15	13	16

The body parts imaged with MRI by age range. H/N: Head/neck. C/A/P: Chest/abdomen/pelvis. Spine: This includes cervical, thoracic, and lumbar spine. UE: Upper extremity. LE: Lower extremity

In the post-MRI questionnaire, seven patients reported pre-MRI pelvic pain. Of these patients, three reported worsening pain post-MRI, with severity ranging from 1/10 to 6/10. Three additional patients reported new onset pelvic pain during or after completion of their MRI, ranging from 1/10 to 2/10. Of the 6 patients that reported new onset pelvic pain (Table 2), two underwent MRI’s of their head/neck and one patient each underwent an MRI of their pelvis, lumbar spine, cervical spine, and lower extremity. Seven patients (including one patient who also reported new onset pelvic pain) reported new onset non-pelvic post-MRI pain. Reported pain included right hand numbness, chest pain, low back pain, right elbow pain, and right knee pain, with severity ranging from 1/10 to 7/10. The remainder of the participants (*n* = 58) reported no new pain.

**Table 2:** Patients that reported new pelvic pain post-MRI and the body region imaged by MRI

Region of the body imaged by MRI	Number of patients
Head/neck	2^a^
Pelvis	1^b^
Lumbar-spine	1^c^
Cervical-spine	1^d^
Lower extremity	1^e^

^a^Two patients reported 1/10 and 5/10 pain. The patient that reported 5/10 pain had reported pelvic pain pre-MRI

^b^One patient reported new 6/10 pain. Of note, this patient had reported pelvic pain pre-MRI

^c^One patient reported 2/10 pain, described as transient and mild cramping that resolved by the time she filled out the questionnaire

^d^One patient reported 1/10 pain, described as a small stinging sensation

^e^One patient reported 2/10 pain, described as pelvic throbbing and discomfort

## Discussion

Cu-IUDs are common devices for long-term, reversible contraception by many women. Currently, these devices are listed as “MR conditional” with multiple previous in-vitro studies demonstrating lack of interaction within the magnetic field [1, 16, 21]. To our knowledge, this is the first in-vivo prospective study evaluating the position of a Cu-IUD both before and after a 3 T MRI examination and pelvic pain after MRI. There is only one reported case of dislocation and partial uterine perforation described after undergoing a 3 T MRI exam with a Cu-IUD, although the authors acknowledged that the IUD may have been perforated prior to the MRI [17]. It is also worth noting that the displaced IUD in this study was the NOVA T 380 device, which contains a silver core compared to our studied Paragard T380A, which has a plastic core [1, 23]. Both devices have 380 mm^2^ of copper surface area.

Here, our study demonstrated zero cases (out of 70) of displaced Cu-IUDs following 3 T MRI examinations in women with appropriately positioned IUDs confirmed on pre-MRI ultrasound. This suggests that these examinations can be performed safely at 3 T MRI for this patient population. Our findings are in line with a prior in-vitro study that demonstrated no safety concerns regarding the use of Cu-IUDs at 3 T, even at a whole body SAR of 4 W/kg, double what the current MRI conditions are set for Paragard [15]. The authors went on to state that there is no justification to routinely check the Cu-IUD placement following MRI examination. A second in-vitro study in 2018 using a tissue-mimicking phantom concluded that patients with implanted Cu-IUDs can safely undergo MRI [24]. These studies corroborate well with our in-vivo results.

Interestingly, 6 of 70 patients reported new mild or moderate pelvic pain during or after the completion of their MRI, ranging from 1/10 to 6/10. A previous ex-vivo study demonstrated that, within a 3 T MRI (Ingenia, Philips Healthcare, Best, the Netherlands), Cu-IUDs deflected up to 0.5 ± 0.5° and experienced temperature increases of 2.4 °C (at 3 T SAR 2 W/kg) and of 4.8 °C (at 3 T SAR 4 W/kg) [25]. A subsequent study of Cu-IUDs in a tissue-mimicking phantom demonstrated no significant increase in temperature, torque, angulation, nor translational motion at 3 T [24]. It should be noted though that these researchers did not explicitly use the Paragard T380A, but rather the Nova T as well as a similar, but smaller, European-approved device manufactured by Mona Lisa N.V. [24, 25]. It is possible that similar heating characteristics may have contributed to our patient’s new onset pelvic pain following 3 T MRI, although this is entirely speculation. Potential future studies could investigate the correlation of symptomatic pelvic pain when controlling for different body regions being imaged (e.g. uterus within the field of view versus extremity imaging, uterus within the main magnetic field versus gradient field), as well as patient follow-up to evaluate the duration, resolution, and complications associated with any new pelvic pain identified during or after MRI.

The primary limitation of this study is the sample size. This study started pre-COVID19 and was halted due to COVID19-related regulations on prospective clinical research in 2020–2021 and lack of ultrasound and MRI research time after 2021 due to technologist shortages. A larger study would improve the statistical reliability. Additionally, this is a single-site prospective study using a single MRI manufacturer, MRI model, and single Cu-IUD model (Paragard T380A), which may limit the generalizability of our findings. Third, the body part imaged was not standardized, however this allowed for more generalizability as patients with Cu-IUDs have MRI examinations on many different body parts in clinical practice. Finally, with no events of displacement out of 70 patients, it is difficult to prove that an event will not happen had more patients been enrolled. However, our statistical analysis shows that it is unlikely that an event like Cu-IUD displacement would happen in the 3 T environment under the prescribed MRI conditions.

Accepting these limitations, the results of our prospective in-vivo study show that zero out of 70 patients had IUD displacement after undergoing 3 T MRI using low SAR settings. These findings support the current standard of care that does not recommend patients who have a copper-containing IUD to have a routine ultrasound before or after an MRI, unless new or worsening pelvic pain develops. It is our hope that this study adds to the growing evidence that 3 T MRI can be performed safely for patients with Cu-IUDs, empowering ordering providers and radiologists with the confidence to perform these examinations for this patient population.
